# Cyproterone acetate enhances TRAIL-induced androgen-independent prostate cancer cell apoptosis via up-regulation of death receptor 5

**DOI:** 10.1186/s12885-017-3153-4

**Published:** 2017-03-07

**Authors:** Linjie Chen, Dennis W. Wolff, Yan Xie, Ming-Fong Lin, Yaping Tu

**Affiliations:** 1 Department of Pharmacology, Creighton University School of Medicine, 2500 California Plaza, Omaha, NE 68178 USA; 2 Department of Biomedical Sciences, University of South Carolina School of Medicine Greenville, Greenville, SC USA; 3 Department of Biochemistry and Molecular Biology, University of Nebraska Medical Center, Omaha, NE 68198 USA

**Keywords:** Apoptosis, Castration-resistant prostate cancer, CHOP, Cyproterone acetate, Death receptor 5, Endoplasmic reticulum stress, PARP cleavage, TRAIL, PC-3, DU145

## Abstract

**Background:**

Virtually all prostate cancer deaths occur due to obtaining the castration-resistant phenotype after prostate cancer cells escaped from apoptosis and/or growth suppression initially induced by androgen receptor blockade. TNF-related apoptosis-inducing ligand (TRAIL) was an attractive cancer therapeutic agent due to its minimal toxicity to normal cells and remarkable apoptotic activity in tumor cells. However, most localized cancers including prostate cancer are resistant to TRAIL-induced apoptosis, thereby creating a therapeutic challenge of inducing TRAIL sensitivity in cancer cells. Herein the effects of cyproterone acetate, an antiandrogen steroid, on the TRAIL-induced apoptosis of androgen receptor-negative prostate cancer cells are reported.

**Methods:**

Cell apoptosis was assessed by both annexin V/propidium iodide labeling and poly (ADP-ribose) polymerase cleavage assays. Gene and protein expression changes were determined by quantitative real-time PCR and western blot assays. The effect of cyproterone acetate on gene promoter activity was determined by luciferase reporter assay.

**Results:**

Cyproterone acetate but not AR antagonist bicalutamide dramatically increased the susceptibility of androgen receptor-negative human prostate cancer PC-3 and DU145 cells to TRAIL-induced apoptosis but no effects on immortalized human prostate stromal PS30 cells and human embryonic kidney HEK293 cells. Further investigation of the TRAIL-induced apoptosis pathway revealed that cyproterone acetate exerted its effect by selectively increasing death receptor 5 (DR5) mRNA and protein expression. Cyproterone acetate treatment also increased DR5 gene promoter activity, which could be abolished by mutation of a consensus binding domain of transcription factor CCAAT-enhancer-binding protein homologous protein (CHOP) in the DR5 gene promoter. Cyproterone acetate increases CHOP expression in a concentration and time-dependent manner and endoplasmic reticulum stress reducer 4-phenylbutyrate could block cyproterone acetate-induced CHOP and DR5 up-regulation. More importantly, siRNA silencing of CHOP significantly reduced cyproterone acetate-induced DR5 up-regulation and TRAIL sensitivity in prostate cancer cells.

**Conclusions:**

Our study shows a novel effect of cyproterone acetate on apoptosis pathways in prostate cancer cells and raises the possibility that a combination of TRAIL with cyproterone acetate could be a promising strategy for treating castration-resistant prostate cancer.

**Electronic supplementary material:**

The online version of this article (doi:10.1186/s12885-017-3153-4) contains supplementary material, which is available to authorized users.

## Background

Prostate cancer is the most common non-skin malignancy in males in developed countries, and the second most frequent cause of male cancer-related death [1]. Androgen deprivation/castration is the principal option for treating prostate cancer. Cyproterone acetate (CPA), a synthetic steroid was initially utilized for prostate cancer treatment because of its ability to block the androgen receptor (AR) and reduce serum testosterone levels. CPA has shown favorable results when used as androgen deprivation monotherapy for advanced prostate cancer [2] and continues to be a drug of interest to oncologists treating prostate cancer [3]. However, CPA also binds to a range of other steroid receptors [4]. Patients in the US with locally advanced prostate cancer typically receive gonadotropin-releasing hormone inhibitors and/or a nonsteroidal pure anti-androgen such as bicalutamide (Bic) for obtaining maximal androgen blockade with minimal off-target effects. Unfortunately, prostate cancer eventually becomes castration resistant (CR) and proliferates despite continued androgen deprivation therapy [5–7]. A major challenge in the field of prostate cancer research is to develop more efficacious treatments for CR prostate cancer.

Dysregulation of apoptosis in cancer contributes to uncontrolled cell growth and is a hallmark of malignancy. Consequently, reactivating and/or triggering apoptosis in cancer cells is a frequent goal of new anticancer therapies. TRAIL is a promising cancer therapeutic agent due to its minimal toxicity to normal cells and remarkable apoptotic activity in cancer cells [8–10]. Upon binding to its death receptor 5 (DR5), TRAIL induces the formation of the Death Inducing Signaling Complex (DISC) that includes DR5, the adaptor molecule FADD and pro-caspase-8. This causes the activation of caspase-8, leading to the activation of caspase-9 and subsequently activation of the executioner caspases (caspase-3, −6 and −7) [11–13]. These latter caspases degrade substrates such as poly (ADP-ribose) polymerase (PARP) to cause apoptosis. Several phase II clinical cancer therapy trials were conducted in which either recombinant TRAIL or monoclonal antibodies that have a longer half-life and selectively activate DR5 were administered to patients with cancers expected to respond to this therapy. These drugs were well tolerated but their benefits were modest and typically did not achieve statistical significance of efficacy [14–17], leading to a generalized conclusion that primary cancers are TRAIL-resistant.

Similar to localized in situ tumors, various prostate cancer cell lines are also resistant to TRAIL-induced apoptosis [18]. During the course of preliminary experiments with prostate cancer cell lines PC-3 and DU145, we serendipitously noted an unexpected finding that the sensitivity of these androgen-independent cells to TRAIL was markedly enhanced when the cells had been pretreated with CPA. Our further study demonstrated that CPA enhances TRAIL-induced apoptosis in AR-negative, androgen-independent prostate cancer cells by up-regulation of DR5. This up-regulation occurs after induction of transcription factor CCAAT-enhancer-binding protein homologous protein (CHOP) protein, which then binds to the promoter of the DR5 gene to increase its expression. To the best of our knowledge, our results are the first to show this effect of CPA on TRAIL-induced prostate cancer cell apoptosis, and raise the possibility that a combination of TRAIL with CPA for its associated efficacy unrelated to androgen antagonism could improve treatment of CR prostate cancer.

## Methods

### Cell lines and culture

DU145 (ATCC® HTB-81™), PC-3 (ATCC® CRL-1435™) and HEK293 (ATCC® CRL-1573™) cell lines were from the American Type Culture Collection (Manassas, VA). Immortalized human prostate stromal PS30 cells were kindly provided by Dr. Debra Schwinn (Duke University Medical Center) [19]. DU145 and HEK293 cells were cultured in DMEM medium with 10% fetal bovine serum (FBS); PC-3 and PS30 cells were cultured in RPMI 1640 medium with 10% FBS. Before treatment with compounds, FBS in culture medium was reduced to 2%. CPA, Bic, cycloheximide and 4-phenylbutyrate (4-PBA) were purchased from Sigma-Aldrich (St. Louis, MO). Recombinant TRAIL was a gift from Dr. Xu Luo (University of Nebraska Medical Center) [20].

### Apoptosis assay

The ApopNexin™ FITC Apoptosis Detection Kit (Millipore, Billerica, MA) was used for the annexin V/propidium iodide (PI) flow cytometry analysis of early apoptosis by FACSCaliber cytometry (BD Biosciences) at the Creighton University Flow Cytometry Core Facility. PARP antibody (#9532, Cell Signaling Technology, Beverly, MA) was used to detect cleavage of PARP for the western blot method of apoptosis detection. The percentage of cleaved PARP (89 kDa) in total PARP (116 kDa full length plus cleaved PARP) was used as indicator of apoptosis.

### Western blot assay

Protein extracts from cultured cells were quantified using Pierce™ Coomassie (Bradford) Protein Assay Kit (Thermo Fisher Scientific, Waltham, MA) and then subjected to western blot analysis as we reported [21]. Primary antibodies against DR5 (#AB16942, Milllipore), caspase-8 (#9746, Cell Signaling Technology), FLIP (#3210, Cell Signaling Technology), FADD (#610399, BD Transduction Laboratories™), CHOP (#2895, Cell Signaling Technology), β-actin (Santa Cruz Biotechnology) and Bid (kindly provided by Dr. Xu Luo) [20] were used to detect relevant proteins of interest.

### Quantitative real-time PCR

Total RNA extraction and quantitative real-time PCR were conducted as described [22]. The DR5, DR4 and β-actin primers are listed in Additional file 1: Table S1.

### Construction of plasmids

All primers are shown in Additional file 1: Table S1. The DR5 promoter fragments (−711/+3) were amplified from DU145 cell genomic DNA by PCR with BglII- and HindIII-flanked primers and cloned into the pGL3-Basic luciferase reporter vector (Promega, Fitchburg, WI). Progressive deletion mutants of the DR5 promoter were created by PCR with promoter-specific primers, using the DR5 promoter luciferase plasmid (−711/+3) as a template. The DR5 luciferase plasmid DR5-P(mtCHOP) with mutations in the CHOP binding site was generated by over-lap PCR. Nucleotide changes are underlined. All constructs were validated by DNA sequencing.

### Plasmids transfection and luciferase reporter assay

DU145 cells (1.6 × 10^5^ cells) were co-transfected with 100 ng of the Renilla luciferase plasmid pRL-SV40P (Addgene) and 400 ng of firefly luciferase constructs containing the DR5 promoter region using Lipofectamine 2000 (Life Technologies). After 24 h, the cells were treated with CPA or solvent for an additional 24 h. A Dual-Glo® Luciferase Assay kit (Promega) and Sirius luciferase assay system (Berthold, Germany) were used to detect luciferase activity and the firefly luciferase activity was normalized by Renilla luciferase activity.

### Small interference RNA (siRNA) transfection

CHOP siRNA (#L-004819-00-0005) and control siRNA (#D-001810-01-05) were purchased from GE Dharmacon (Lafayette, CO). DU145 cells were transfected with 300 nM siRNA using Nucleofector® II Device (Lonza, Walkersville, MD) according to manufacturer’s instructions. 24 h later, cells were subjected to treatment with the indicated compounds.

### Data statistical analysis

Data are expressed as means ± SEM of at least three determinations. Groups were compared using Student’s *t* test for unpaired observations or two-way ANOVA with the Bonferroni correction for multiple comparisons. * *p* < 0.05; ** *p* < 0.01; ns, not significant.

## Results

### CPA enhances TRAIL-induced apoptosis in androgen-independent prostate cancer cells

**Fig. 1:**
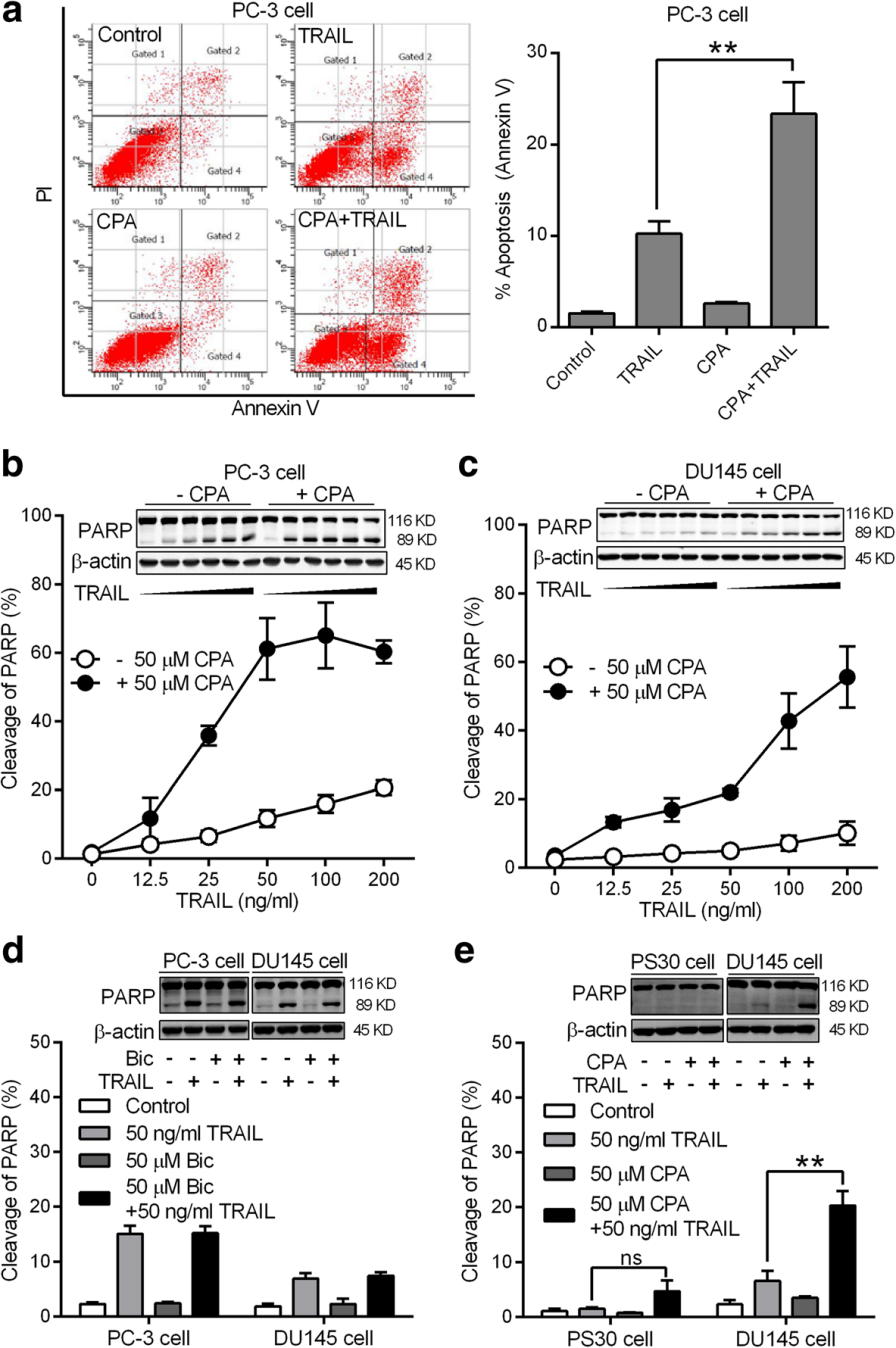
CPA sensitized AR-negative androgen-independent prostate cancer cells to TRAIL-induced apoptosis. **a** PC-3 cells were pretreated without (control) or with 50 μM CPA for 24 h, and then treated without or with 50 ng/ml TRAIL for 6 h. Both suspended and attached cells were harvested. Apoptosis was measured by the annexin V/PI flow cytometry method as described in Materials and Methods. Left panel, representative flow cytometry histograms of apoptosis assay; right panel, statistical analysis of results from three independent experiments. Data are means ± S.E. with **p < 0.01. PC-3 cells **b** and DU145 cells **c** were pretreated without or with 50 μM CPA for 24 h, and then treated with indicated concentrations of TRAIL for 6 h. **d** PC-3 and DU145 cells were pretreated without (control) or with 50 μM Bic for 24 h, and then treated without or with 50 ng/ml TRAIL for 6 h. **e** PS30 and DU145 cells were pretreated without or with 50 μM CPA for 24 h, and then treated without or with 50 ng/ml TRAIL for 6 h. Cleavage of PARP was measured by western blot analysis. Data shown are means ± S.E. (*n* = 3) with **p < 0.01, ns: not significant. Insets: Representative western blot images of PARP and β-actin

PC-3 and DU145 are two androgen-independent prostate cancer cell lines and also often characterized as AR negative. We first examined the effect of CPA treatment on TRAIL-induced apoptosis in these two cell lines. Using the annexin V/PI assay method, we found that CPA (50 μM) alone only slightly increased apoptosis of PC-3 cells in the absence of TRAIL, while significantly enhanced TRAIL-induced cell apoptosis by 2.5-fold (Fig. 1a). Western blot analysis of PARP cleavage confirmed that CPA treatment not only increased the maximum PARP cleavage by 3-fold but also reduced the TRAIL concentration needed to induce 50% of maximal cleavage of PARP (the EC_50_) from 50 ng/ml to 20 ng/ml (Fig. 1b). DU145 cells are highly resistant to TRAIL-induced apoptosis with only a marginal increase in PARP cleavage observed even in the presence of 100 ng/ml TRAIL. Pretreatment of CPA (50 μM) sensitized DU145 cells to TRAIL-induced apoptosis (Fig. 1c). At 100 ng/ml TRAIL, PARP cleavage was increased from 5 to 40% by CPA pretreatment.

However, another classical AR antagonist Bic (50 μM) had no effects on TRAIL induced cleavage of PARP in PC-3 and DU145 cells (Fig. 1d). As expected, immortalized normal human prostate stromal PS30 cells are resistant to TRAIL. Pretreatment with 50 μM CPA had no significant effects on TRAIL (50 ng/ml) induced cleavage of PARP in these prostate cells whereas cleavage of PARP in DU-145 cells were increased by 4-fold (Fig. 1e). Therefore, we focused our studies on DU145 cells.

### Effects of CPA on TRAIL-induced apoptosis are dependent on the activation of caspase-8

**Fig. 2:**
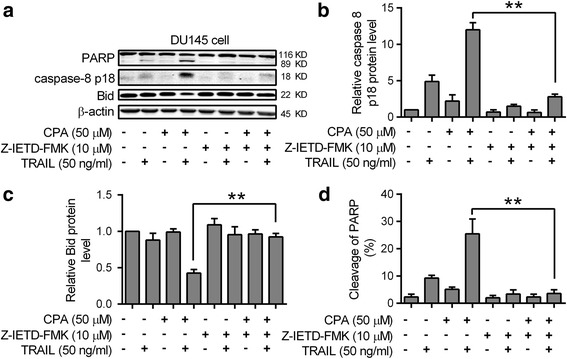
Effects of CPA on TRAIL-induced apoptosis are dependent on the activation of caspase-8. DU145 cells were pretreated without or with 50 μM CPA for 24 h, and then treated without or with 50 ng/ml TRAIL for 6 h. When needed, 10 μM Z-IETD-FMK was pre-added to cells 1 h before TRAIL treatment. Cells were harvested and subjected to western blot analysis. **a** Representative western blot images of PARP, caspase-8 p18, Bid and β-actin were shown. The western blot results were quantitated and normalized to β-actin. The caspase-8 p18 **b** and Bid **c** protein levels relative to those of control cells and the percentage of cleaved PARP **d** are shown. Data shown are means ± S.E. (*n* = 3) with **p < 0.01

The binding of TRAIL to its DR5 receptor leads to the cleavage and activation of caspase-8, a critical step in the extrinsic pathway for cell apoptosis. As shown in Fig. 2a, CPA enhanced TRAIL-induced production of the p18 fragment of caspase*-*8 in DU145 cells and markedly increased TRAIL-induced cleavage of the BH3-only protein Bid, a critical mediator of the mitochondrial apoptotic pathway. Pretreatment with caspase-8 inhibitor Z-IETD-FMK effectively blocked CPA/TRAIL-induced caspase-8 p18 production by over 90% (Fig. 2b). Importantly, CPA-enhanced TRAIL-induced cleavage of Bid (Fig. 2c) and PARP (Fig. 2d) was also blocked by Z-IETD-FMK pretreatment.

### CPA selectively up-regulates DR5 expression in prostate cancer cells

**Fig. 3:**
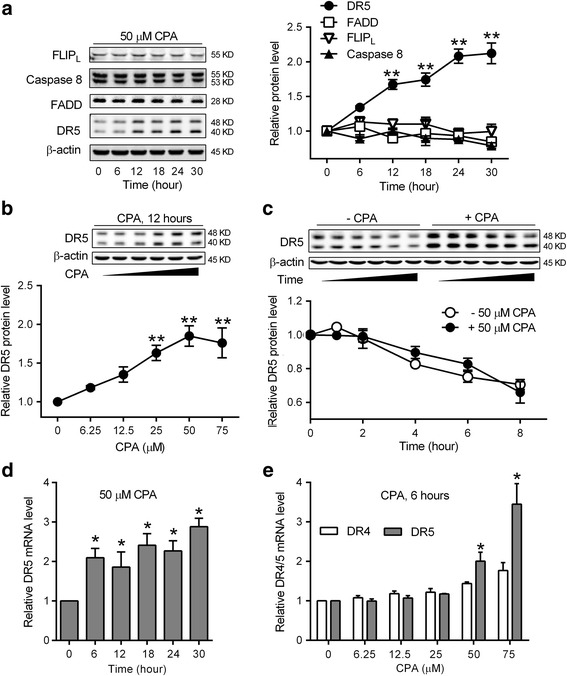
CPA selectively up-regulates DR5 expression in prostate cancer cells. DU145 cells were treated with 50 μM CPA for the indicated time **a** or indicated concentrations of CPA for 12 h **b**. Cells were harvested and subjected to western blot analysis. Data shown are means ± S.E. (*n* = 3) with **p < 0.01. β-actin was used as a loading control and the indicated protein level in control cells without CPA treatment was normalized as one. (**a**, *left panel*) and (**b**, *inset*): Representative western blot images of indicated proteins. **c** DU145 cells were pretreated without or with 50 μM CPA for 24 h, and then treated with 50 μM cycloheximide for 10 min (time = 0). Cells were harvested at indicated times and subjected to western blot analysis. Data shown are means ± S.E. (*n* > 3) with **p < 0.01. DR5 protein levels at 0 h were set as one. Inset: Representative western blot images of DR5 and β-actin. **d** and **e** DU145 cells were treated with 50 μM CPA for the indicated time **d** or indicated concentrations of CPA for 6 h **e**. DR4 and DR5 mRNA levels were determined by quantitative RT-PCR analysis. β-actin was used as an internal control. Data shown are means ± S.E. (*n* = 3) with *p < 0.05

We next examined the components of the DISC and its negative regulator FLIP [23] in DU145 cells for illustrating the potential regulation by CPA. While FLIP and other DISC components (caspase-8 and FADD) did not show a change, DR5 protein was elevated following CPA treatment in a time- and concentration-dependent manner (Fig. 3a, b).

We further determined if the elevated DR5 protein level seen with CPA treatment was due to a prolonged DR5 protein half-life. DU145 cells without or with CPA-induced DR5 elevation were incubated with cycloheximide to block new protein biosynthesis. The disappearance of DR5 was followed over time by western blot. Figure 3c shows a representative western blot indicating that independent of CPA treatment, DR5 was gradually degraded with a half-life of at least 8 h and CPA treatment had no significant effects on DR5 degradation.

We also carried out quantitative RT-PCR analysis to investigate DR5 mRNA changes induced by CPA treatment. As shown in Fig. 3d, CPA increased DR5 mRNA expression by approximately 2-fold at 6 h and this induction was persistent over the ensuing 24 h. In addition, CPA increased DR5 mRNA levels, but not DR4 mRNA levels, in a concentration-dependent manner (Fig. 3e).

### CPA stimulates DR5 promoter activity via a CHOP binding motif in prostate cancer cells

**Fig. 4:**
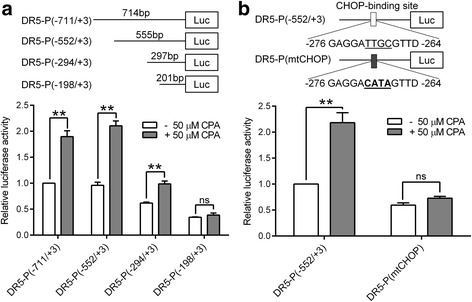
The CHOP binding domain is critical for CPA-activated DR5 gene promoter activity. DU145 cells were transfected with the luciferase reporter plasmids containing various lengths of the DR5 gene promoter. Cells were treated without or with 50 μM CPA for 24 h and then subjected to luciferase activity assays. **a** Upper panel, schematic structure of reporter plasmids; lower panel, statistical analysis of three independent luciferase assay results. Data shown are means ± S.E. with **p < 0.01, ns: not significant. The relative luciferase activity of DR5-P(−711/+3) without CPA treatment was set as one. **b** Upper panel, schematic structure of DR5 promoter reporter plasmids containing wide-type or mutated CHOP binding site; lower panel, statistical analysis of three independent luciferase assay results. Data shown are means ± S.E. with **p < 0.01, ns: not significant. The relative luciferase activity of DR5-P(−552/+3) without CPA treatment was set as one

DR5 proximal promoter containing a 714-bp DNA fragment upstream of the human DR5 coding region was amplified using the genomic DNA from DU145 cells as a template, cloned into the luciferase reporter vector pGL3-Basic, and designated as DR5-P(−711/+3) (the “+3” represents the third base from the putative transcription start site). Luciferase reporter gene constructs containing the various 5’ flanking regions of the DR5 promoter were also generated (Fig. 4a, upper section). The constructs were transiently transfected into DU145 cells, and their promoter activities were determined in the presence or absence of 50 μM CPA. As shown in Fig. 4a (lower section), following deletion, the DR5-P(−552/+3) plasmid still retains the basal promoter activity, similar to that of the DR5-P(−711/+3) plasmid. CPA treatment increased the promoter activities of DR5-P(−711/+3) and DR5-P(−552/+3) by 1.9- and 2.2-fold, respectively. The DR5-P(−294/+3) plasmid still retains 62% of the DR5-P(−711/+3) basal promoter activity and displayed a similar CPA induction of DR5-P(−711/+3) (1.61- versus 1.9-fold). However, a further 96-bp deletion of DR5-P(−294/+3) resulted in a significant loss of the basal promoter activity (0.34 versus 0.62) and the complete loss of CPA induction, suggesting that this deleted 96-bp fragment (−294/−198) contains key elements of the DR5 promoter that may be responsible for the stimulatory effect of CPA.

Using TFSEARCH, a tool for locating transcription factor binding sites, we identified a consensus transcription factor CHOP binding domain at −276/-264 bp in this 96-bp fragment (Fig. 4b, upper panel). CHOP is reported to be responsible for DR5 up-regulation induced by many drugs [24]. To determine whether the CHOP-binding motif is critical for CPA-stimulated DR5 promoter activity, we mutated the consensus CHOP-binding domain in DR5-P(−552/+3) plasmid. Mutation of this consensus CHOP binding domain significantly reduced the basal promoter activity by 50% and completely abolished CPA induction (Fig. 4b, lower panel). These results demonstrate that this consensus CHOP binding domain (−276/−264) is critical for both basal and CPA-activated DR5 promoter activity.

### Endoplasmic reticulum stress (ER stress) is involved in CPA induced CHOP protein up-regulation in prostate cancer cells

**Fig. 5:**
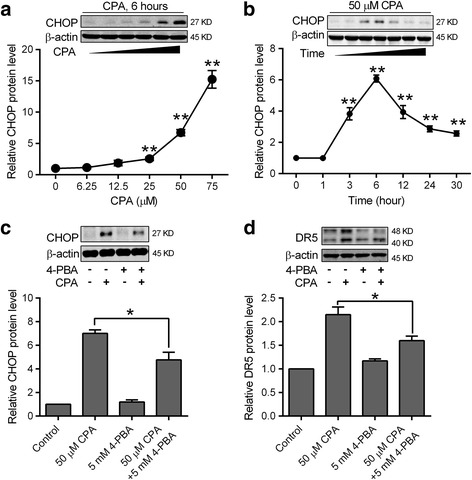
ER stress is involved in CPA induced CHOP protein up-regulation in prostate cancer cells. DU145 cells were treated with the indicated concentrations of CPA for 6 h **a** or 50 μM CPA for the indicated times **b. c** and **d** DU145 cells were pretreated with 5 mM ER stress reducer 4-PBA for 1 h, and then treated with 50 μM CPA for 6 h **c** or 30 h **d**. Cells were harvested and subjected to western blot analysis. β-actin was used as a loading control and the indicated protein level in control cells without treatment was set as one. Data shown are means ± S.E. (*n* > 3) with * *p* < 0.05, ** *p* < 0.01. Insets: Representative western blot images of CHOP, DR5 and β-actin

We next examined whether CPA increased CHOP protein expression in prostate cancer cells. The basal level of CHOP protein is very low in DU145 cells, but was significantly increased following 6 h treatment with various concentrations of CPA (Fig. 5a). CPA (50 μM) treatment increased CHOP protein expression in a time-dependent manner to a maximal 6-fold induction at the time of 6 h and this induction was gradually reduced but was still 2-fold higher than the basal level at the time of 30 h (Fig. 5b). Since CHOP protein is a marker of ER stress [25], we also investigated whether CPA induced CHOP protein could be blocked by ER stress reducer 4-phenylbutyrate (4-PBA). As shown in Figs. 5c, d, after pretreatment of DU145 cells with 5 mM 4-PBA, CPA induced up-regulation of CHOP and DR5 protein were reduced by 40–50%. Furthermore, we found that neither DR5 protein expression nor TRAIL induced cleavage of PARP was affected by CPA treatment in ER stress resistant HEK293 cells (Additional file 2: Figure S1).

### Up-regulated CHOP contributes to CPA-induced DR5 up-regulation and TRAIL sensitivity in prostate cancer cells

**Fig. 6:**
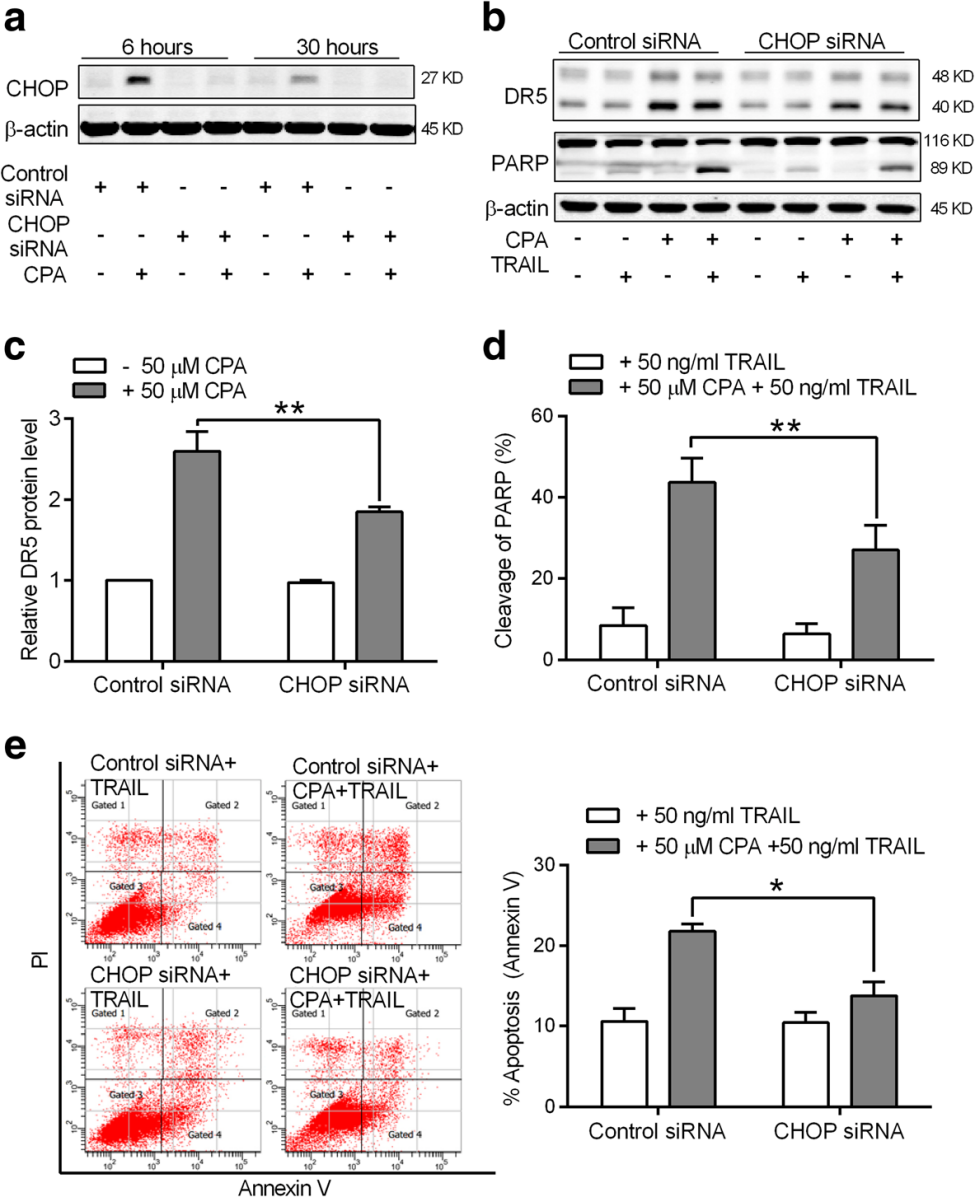
CHOP up-regulation contributes to CPA induced DR5 up-regulation and TRAIL sensitivity in prostate cancer cells. **a** DU145 cells were transfected with control siRNA or CHOP siRNA for 24 h, and then treated without or with 50 μM CPA for 6 and 30 h. Cells were then harvested and subjected to western blot analysis. A representative western blot result for the indicated proteins of three separate experiments is shown. **b**-**e** DU145 cells transfected with control siRNA or CHOP siRNA were treated without or with 50 μM CPA for 24 h, followed by 6 h of treatment without or with 50 ng/ml TRAIL. Cells were then harvested and subjected to western blot analysis. A representative western blot result for the indicated proteins is shown in **b**. The relative DR5 protein level and cleavage of PARP were quantitated and are shown in **c** and **d**, respectively. β-actin was used as a loading control. Data shown are means ± S.E. (*n* > 3) with **p < 0.01. (E) Apoptosis was measured by the annexin V/PI flow cytometry method. Left panel, representative flow cytometry histograms of apoptosis assay; right panel, statistical analysis of results from three independent experiments. Data are means ± S.E. with *p < 0.05

To further determine whether CHOP is responsible for CPA-enhanced DR5 protein expression, DU145 cells were transfected with control siRNA or CHOP-specific siRNA for 24 h, followed by CPA (50 μM) treatment for 6 h. As shown in Fig. 6a, CHOP siRNA largely abolished CPA-induced CHOP expression at the time of 6 and 30 h. Silencing CHOP expression reduced CPA-enhanced DR5 protein expression by about 50% (Fig. 6b, c). More importantly, silencing CHOP expression reduced CPA-stimulated TRAIL-induced PARP cleavage from 40 to 20% (Fig. 6b, d). An annexin V/PI flow cytometry analysis indicated that CPA treatment only increased TRAIL induced cell apoptosis by 1.3-fold in CHOP-silenced DU145 cells as compared 2-fold in control DU145 cells (Fig. 6e). Together, our results suggest that induction of CHOP plays an important role in CPA-enhanced TRAIL-induced prostate cancer cell apoptosis.

## Discussion

TRAIL/DR activation triggers the external apoptosis pathway, which is proposed to be effective against tumors that have acquired resistance to conventional therapy [8, 15, 17]. However, prostate cancer is comparatively resistant to TRAIL-treatment as confirmed in our preliminary studies with prostate cancer cell lines DU145 and PC-3. Thus, the focus has shifted toward developing compounds and/or combination therapies that improve cancer cell susceptibility to TRAIL in a clinical setting [26]. A few small molecule inhibitors or natural compounds were found to enhance the efficacy of TRAIL-induced apoptosis of prostate cancer cells [27]; nevertheless, their mechanism of action and toxicity are typically unknown. To our surprise, we noted that pretreatment of AR-negative, androgen-independent prostate cancer PC-3 and DU145 cells with anti-androgen CPA greatly enhanced their sensitivity to TRAIL.

TRAIL typically activates the extrinsic apoptosis pathway by binding to death receptors. Upon binding to its DR5 receptor, TRAIL induces the activation of caspase-8 that can directly activates caspase-3 via cleavage (extrinsic pathway). In most cancer cells, a limited amount of activated caspase-8 is produced, which is insufficient to directly cleave and activate caspase-3 [28, 29]. Instead, activated caspase-8 may preferentially cleave the cytosolic protein Bid [30], causing release of cytochrome c from mitochondria and the formation of the apoptosome to activate caspase-3 (intrinsic pathway). The present study found that CPA treatment significantly increased TRAIL-induced cleavage of Bid, which was also largely blocked by the pretreatment with caspase-8 inhibitor Z-IETD-FMK. This result is consistent with our recent report that Bid is primarily cleaved by caspase 8 upon TRAIL treatment, which in turn activates the mitochondrial apoptotic pathway, leading to degradation of PARP to cause apoptosis [20]. Thus, CPA could enhance TRAIL-induced cell apoptosis via intrinsic signaling pathways.

Results of further studies show that this enhanced sensitivity to TRAIL is a consequence of DR5 up-regulation, which occurred because CPA induces CHOP protein expression. CHOP protein is a recognized marker of ER stress [25], and the unfolded protein response induced by ER stress is a target of many anticancer drugs in development [31]. ER stress inducer tunicamycin enhances TRAIL induced apoptosis in prostate cancer cells [32]. We found that ER stress reducer 4-PBA significantly attenuates the CPA-induced increases in CHOP and DR5 expression. In addition, CPA treatment had no effects on DR5 expression or TRAIL sensitivity in human embryonic kidney HEK293 cells. Interestingly, HEK293 cells were shown to be comparatively resistant to ER stress [24]. Thus, our available data suggest that ER stress is necessary for the CPA enhancement of TRAIL sensitivity in prostate cancer cells.

CPA represents the first generation of AR blockers for prostate cancer androgen deprivation therapy. Since PC-3 and DU145 cells are AR negative and androgen-independent and a classical AR antagonist Bic had no effects on TRAIL induced cleavage of PARP, it is unlikely that CPA enhances TRAIL sensitivity in PC-3 and DU145 cells via its anti-androgen effects. Interestingly, PC-3 and DU145 cells express functional glucocorticoid receptors (GR) [33] and a recent study showed that GR could substitute for AR function and contributes to CR in prostate cancer [34]. In fact, GR could confer apoptotic resistance to chemotherapy in some solid tumors [35]. Inasmuch as CPA also functions as a GR antagonist [4], this could be contributing to our observed CPA-enhancement of TRAIL-induced apoptosis. We are currently investigating this possibility.

The desire for more robust pure anti-androgens has driven new drug developments for advanced prostate cancer over the past many years. Nonetheless, a drug with multiple targets can sometimes be more effective than a pure drug [36]. Our data with synthetic steroid CPA indicate that the inclusion of some off-target effect of this drug could potentially improve the efficacy of CR prostate cancer treatment. In contrast to a wide range of other drugs that have been shown to enhance TRAIL sensitivity, CPA is approved for clinical use and still remains widely used in many countries. Finding new tricks for an old drug such as CPA and related off-patent drugs may represent an efficient pathway for drugs that can be used in combination with TRAIL therapy to better treat CR prostate cancer [37]. The appeal of TRAIL as an anti-cancer agent is due to its selectivity for inducing apoptosis in cancerous tissue. Our data show that CPA treatment did not augment TRAIL effects in normal human prostate stromal cells and embryonic kidney cells, which suggest that a combination of CPA with TRAIL is a potential therapeutic strategy without severe side effects in treating prostate cancer patients.

It should be noted that CPA itself was reported to have a dose-related hepatotoxicity [2], so regular hepatic function tests may still be necessary for the patients with a combination of CPA with TRAIL therapy. Intermittent treatment with CPA for locally advanced and metastatic prostate cancer was reported to maintain efficacy while toxicity and costs were reduced [38]. Given our data, it seems plausible that adding TRAIL to CPA therapy could also help curtail the dose-related toxicity. Thus, further characterization of the precise mechanisms by which CPA induces susceptibility to TRAIL-mediated apoptosis may aid with the development of agents possessing greater long-term efficacy for the treatment of CR prostate cancer.

## Conclusions

**Fig. 7:**
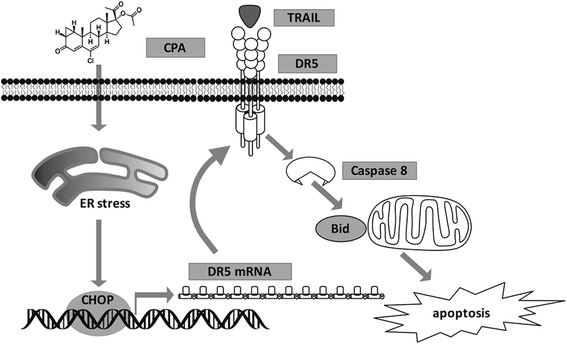
Schematic diagram of the mechanism for CPA enhancement of TRAIL induced apoptosis in prostate cancer cells. CPA treatment induces CHOP protein expression via ER stress response pathways. CHOP protein binds to the promoter region of DR5 gene and up-regulates DR5 mRNA and protein expression. Up-regulated DR5 protein arguments TRAIL induced Bid-dependent apoptotic pathways in prostate cancer cells

Our study demonstrated for the first time that CPA enhances TRAIL-induced apoptosis in AR-negative, androgen-independent prostate cancer cells via DR5 up-regulation. Moreover, we found that ER stress-dependent induction of transcription factor CHOP contributes to this DR5 up-regulation and apoptosis-enhanced effect (Fig. 7). Our results therefore raise the possibility that a combination of TRAIL with CPA for its associated efficacy unrelated to androgen antagonism could be a promising strategy for improving treatment of CR prostate cancer.

## Additional files

Additional file 1: Table S1. Primer pairs for RT-PCR or construction of plasmids. (XLSX 11 kb)
Additional file 2: Figure S1. CPA treatment had no effects on DR5 expression or TRAIL sensitivity in human embryonic kidney HEK293 cells. Cells were pretreated with or without 50 μM CPA for 24 h, and then treated with or without 50 ng/ml TRAIL for 6 h. Cells were harvested and subjected to western blot analysis of DR5 expression and cleavage of PARP. β-actin was used as a loading control. Data shown are means ± S.E. (n = 3). Inset: Representative western blot images of PARP, DR5 and β-actin. (JPG 475 kb)

## Abbreviations

AR: Androgen receptor
CPA: Cyproterone acetate
Bic: Bicalutamide
CHOP: CCAAT-enhancer-binding protein homologous protein
CR: Castration resistant
DR: Death receptor
DISC: Death Inducing Signaling Complex
EC_50_: TRAIL concentration needed to induce 50% of maximal cleavage of PARP
ER: Endoplasmic reticulum
FBS: Fetal bovine serum
PARP: Poly (ADP-ribose) polymerase
4-PBA: 4-phenylbutyrate
PI: Propidium iodide
TRAIL: TNF-related apoptosis-inducing ligand
