# Supplementary figures and images for: Cyproterone acetate enhances TRAIL-induced androgen-independent prostate cancer cell apoptosis via up-regulation of death receptor 5
Source: BMC Cancer. 2017 Mar 7;17:179. doi: 10.1186/s12885-017-3153-4 (PMC5341373; doi:10.1186/s12885-017-3153-4)

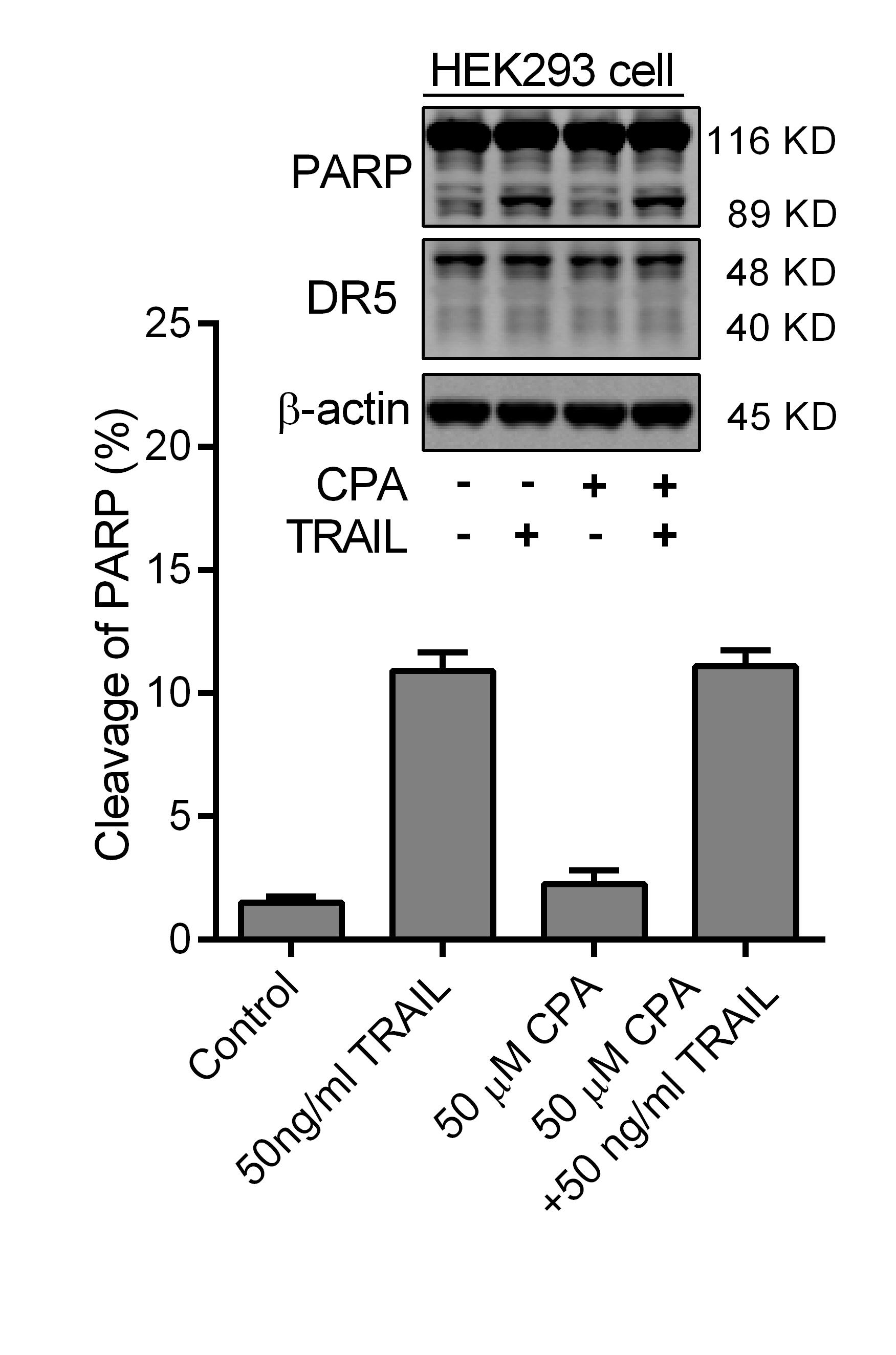

Supplement: Additional file 2: Figure S1. — CPA treatment had no effects on DR5 expression or TRAIL sensitivity in human embryonic kidney HEK293 cells. Cells were pretreated with or without 50 μM CPA for 24 h, and then treated with or without 50 ng/ml TRAIL for 6 h. Cells were harvested and subjected to western blot analysis of DR5 expression and cleavage of PARP. β-actin was used as a loading control. Data shown are means ± S.E. (n = 3). Inset: Representative western blot images of PARP, DR5 and β-actin. (JPG 475 kb) [file 12885_2017_3153_MOESM2_ESM.jpg]
